# Editorial: Child-to-Parent Violence: Challenges and Perspectives in Current Society

**DOI:** 10.3389/fpsyg.2021.699072

**Published:** 2021-06-23

**Authors:** Lourdes Contreras, M. Carmen Cano-Lozano, Francisco Javier Rodríguez-Díaz, Melanie Simmons

**Affiliations:** ^1^Department of Psychology, University of Jaén, Jaén, Spain; ^2^Faculty of Psychology, University of Oviedo, Oviedo, Spain; ^3^Centre for Forensic Behavioural Science, Swinburne University of Technology, Hawthorn, VIC, Australia

**Keywords:** child-to-parent violence, adolescents, family violence, assessment, young offenders, psychosocial variables

Violence committed by young people is a major concern in modern society, with regular media reports about juvenile violence in various contexts and relationships. In the last decade, rates of child-to-parent violence (CPV) have risen dramatically, becoming a significant social problem in some countries. For instance, the Spanish Prosecutor's Office (2020) revealed that police reports of CPV increased by 8% from 4,665 cases in 2017 to 5,055 cases in 2019. This increased awareness of CPV in Spain has resulted in a greater investment in CPV research.

This Research Topic aimed to advance our understanding of CPV across different cultures and populations. It considered the perspectives of both parents and children and comprises one theoretical review and eight original research papers that consider the measurement of CPV and factors related to the development and maintenance of this violent behaviour.

Drawing upon previous definitions used to describe CPV in research, Ibabe developed a theoretical definition of CPV and a typology which described four types, which differ according to the level of coercion and nature of the violence. This review also evaluated eleven instruments that measure CPV according to the Consensus-based Standards for the Selection of Health Measurement Instruments guidelines. Ibabe concluded that the Child-to-Parent Violence Questionnaire (CPV-Q) was the leading instrument in the field, with an additional three instruments showing promise.

Ibabe's research highlighted that most CPV instruments are solely designed to be used with adolescents. However, examining parents' perspectives on the frequency and nature of CPV is critical to understanding this phenomenon. Contreras et al. validated the Child-to-Parent Violence Questionnaire—Parents' version (CPV-Q-P) in a large sample of parents of adolescents. The findings suggested that the measure had strong psychometric properties, and that participants reported that their children frequently engaged in CPV behaviours. Parents reportedly attributed their children's CPV behaviours to instrumental reasons instead of viewing CPV as the result of impulsive emotional reactions.

Seven papers considered factors related to CPV. Martínez-Ferrer et al. explored the relationship between CPV, psychological distress and self-perception within familial and social contexts among adolescents recruited from Mexican schools. They found that the adolescents who engaged in CPV showed higher levels of psychological distress and suicidal ideation, and poorer self-concept within familial and social contexts than non-aggressor adolescents. The authors also found that, although boys were more frequently involved in CPV, girls who were violent showed greater adjustment difficulties. Similarly, Seijo et al. also found that Spanish adolescents displayed poorer psychological, personal, and educational adjustment than non-aggressors, which was related to both victimisation by parents and CPV. Adolescents who engaged in CPV also reported greater strictness and supervision from their parents, with those who were violent towards their mothers reporting low parental warmth.

Although previous studies have noted the importance of individual, family and social variables in CPV, these relationships are complex. Cano-Lozano et al. used structural equation modelling in a large sample of Spanish adolescents to further explore the relationship between parental warmth and CPV through cognitive (hostile attribution), emotional (anger), and social (deviant peers and drug use) variables. They found that the lack of perceived parental warmth was related to hostile attribution and anger. The combination of these factors was associated with an increase in the frequency of reactive CPV behaviours. Perceived parental criticism-rejection was also related to a greater likelihood of associating with deviant peers and drug use, which increased the likelihood of both reactive and instrumental CPV.

Suárez- Relinque et al. examined CPV within a large sample of Mexican adolescents recruited from schools. The authors considered factors such as the problematic use of social networking sites and perceived non-conformist social reputation that have been studied in the broader adolescent violence literature, but have scarcely been examined in the context of CPV. Results suggested that psychological distress, social media usage, non-conformist reputation, and problematic communication with parents were related to CPV.

Three studies explored individual and family variables related to CPV among justice-involved youth. Hernández et al. explored the differences between CPV offenders, non-CPV offenders, and non-offending adolescents. They found that both groups of offenders reported higher frequency of drug use and lower academic performance compared to the non-offender group. Furthermore, CPV offenders reported poorer perceptions of themselves within their families and higher rates of exposure to violence at home when compared to the other groups. Fandiño et al. explored whether individuals who engaged in CPV displayed deficits in psychological adjustment and executive functioning. The results suggested that, compared to the psychometric test norms, young offenders who engaged in CPV displayed significantly higher psychological maladjustment, clinical deterioration, and deficits in executive cognitive functioning. Finally, Vecina et al. explored whether young offenders who were violent towards their parents differed compared to those who were violent towards their partners on five moral foundations (care, fairness, loyalty, authority, and purity). Disregard for authority was the only foundation that independently differentiated CPV offenders from those who were violent against their partners, as CPV offenders were more likely to have negative attitudes towards authority. CPV offenders were also more likely to justify their violence and perceive themselves as aggressive compared to the partner violence group.

This Research Topic generated new theoretical and empirical knowledge on CPV, yielding significant contributions to this field and addressing important issues such as its conceptualisation and assessment. It provided an integrated definition and developed a typology of CPV to delineate four types of CPV behaviour. Additionally, it described the validation of a new measure that can be completed by parents and provided valuable information for researchers and clinicians regarding the evidence base related to the assessment of CPV.

Although the relationship between CPV and parenting practises has previously been demonstrated, this Research Topic enhanced our understanding of the mechanisms underlying this relationship, while also exploring novel variables that have not been previously considered in relation to CPV (i.e., problematic use of social networks sites, perceived non-conformist social reputation, executive functioning and moral foundations). In line with the Socio-Ecological Model, these articles highlight the importance of the interactions between factors from the individual, familial, and social domains to explain this type of violence. Future studies should continue investigating the mechanisms of effect to strengthen our understanding and formulation of CPV behaviour while also identifying ways that we can intervene to either prevent or stop a pattern of CPV from developing.

## Author Contributions

All authors listed have made a substantial, direct and intellectual contribution to the work, and approved it for publication.

## Conflict of Interest

The authors declare that the research was conducted in the absence of any commercial or financial relationships that could be construed as a potential conflict of interest.

